# New Insights into the Molecular Bases of Familial Alzheimer’s Disease

**DOI:** 10.3390/jpm10020026

**Published:** 2020-04-19

**Authors:** Valeria D’Argenio, Daniela Sarnataro

**Affiliations:** 1 CEINGE-Biotecnologie Avanzate scarl, via G. Salvatore 486, 80145 Naples, Italy; 2 Department of Human Sciences and Quality of Life Promotion, San Raffaele Open University, via di val Cannuta 247, 00166 Rome, Italy; 3 Department of Molecular Medicine and Medical Biotechnology, Federico II University, via S. Pansini 5, 80131 Naples, Italy

**Keywords:** Alzheimer’s disease, *APP* mutations, *APOE* alleles, *PSEN1*, *PSEN2*, germline mutations, late onset AD, early onset AD, familial AD, genetics of AD

## Abstract

Like several neurodegenerative disorders, such as Prion and Parkinson diseases, Alzheimer’s disease (AD) is characterized by spreading mechanism of aggregated proteins in the brain in a typical “prion-like” manner. Recent genetic studies have identified in four genes associated with inherited AD (amyloid precursor protein-*APP*, Presenilin-1, Presenilin-2 and Apolipoprotein E), rare mutations which cause dysregulation of APP processing and alterations of folding of the derived amyloid beta peptide (Aβ). Accumulation and aggregation of Aβ in the brain can trigger a series of intracellular events, including hyperphosphorylation of tau protein, leading to the pathological features of AD. However, mutations in these four genes account for a small of the total genetic risk for familial AD (FAD). Genome-wide association studies have recently led to the identification of additional AD candidate genes. Here, we review an update of well-established, highly penetrant FAD-causing genes with correlation to the protein misfolding pathway, and novel emerging candidate FAD genes, as well as inherited risk factors. Knowledge of these genes and of their correlated biochemical cascade will provide several potential targets for treatment of AD and aging-related disorders.

## 1. Introduction

Alzheimer’s disease (AD) is responsible for about 60–70% of cases of dementia, equivalent to an estimated population of 40–50 million persons worldwide. This more than doubled from 1990 to 2016 [1]. Typically, AD is featured by a progressive neurodegeneration that lead to a gradual loss of memory and alterations affecting other cognitive functions, such as spatial cognition, reasoning, word-finding, judgment and problem-solving [2]. Aging is the most important AD risk factor and, even if it may occur at any age, more often AD appears in older individuals (later than 65 years of age) [3,4]. However, early onset AD cases, characterized by the occurrence of clinical signs between 30 and 65 years old, have been also reported [3,4]. Even if atypical phenotypes have been described, clinical and pathological features seem to be the same between early and late onset AD, so that it may be difficult to distinguish these 2 groups [4]. Interestingly, while late onset AD is usually sporadic and doesn’t show any segregation within the families, it has been highlighted that early onset AD is featured by a high recurrence rate within the affected families, thus suggesting the presence of inherited forms of AD [3].

**Figure 1:**
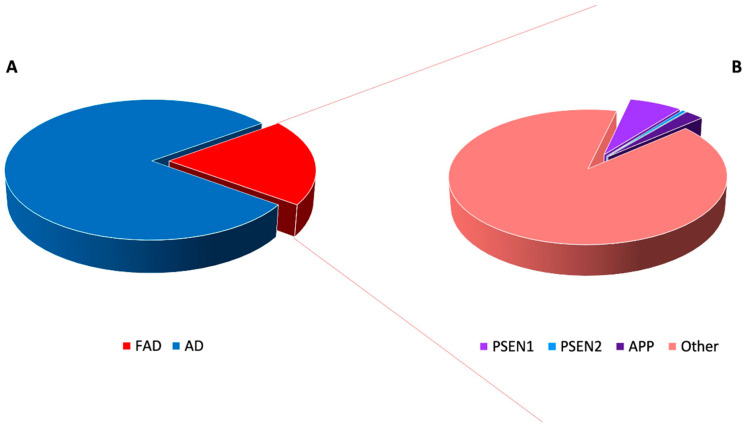
Prevalence and genetic causes of familial Alzheimer’s disease (FAD). FAD represents only a small fraction (about 20%) of all Alzheimer’s disease (AD) cases (**A**). In addition, within FAD, mutations the amyloid precursor protein (*APP*), presenilin 1 (*PSEN1*) or presenilin 2 (*PSEN2*) genes account for a small proportion of cases, underlying that the molecular bases of the larger fraction still remain to be unveiled (**B**).

In particular, it has been estimated that 15–25% of total AD accounts for familial Alzheimer’s disease (FAD) (Figure 1) [5].

In this category, it is included the autosomal dominant Alzheimer’s disease (ADAD), associated to the presence of known causative gene mutations, mainly in the amyloid precursor protein (*APP*), presenilin 1 (*PSEN1*) or presenilin 2 (*PSEN2*) genes, that have been described as highly penetrant, disease-causing FAD genes [3,4,5]. However, mutations in these genes are able to explain just a small percentage of all FAD cases, suggesting the existence of other, inherited, disease-predisposing genes [4]. Since FAD typically shows a sequential, clinical progression from pre-dementia to dementia stage, thus it is crucial to recognize and diagnose it before symptoms onset in order to begin treatments as soon as possible [6,7]. As a consequence, a better understanding of FAD molecular bases, i.e., the identification of the causative genes, may ameliorate the management and the clinical outcome of these patients and of their families.

Recent advances in genomics, i.e., the availability of highly performing next generation sequencing (NGS)-based methods to accurately analyze single genes [8,9] or a subset of genes of interest [10,11,12], up to the whole exome [13,14] or the whole genome [13,14], has prompted the study of the molecular bases of human diseases and is a promising tool to discover novel FAD-related genes [4].

Here, we will review the current knowledge regarding the genetic etiology of FAD. In particular, we will focus first on well-established, highly penetrant, FAD-causing genes. In this context, the relationship between mutations affecting AD-related genes and proteins’ trafficking, folding and aggregation properties will be highlighted, with special attention to APP. Next, novel, emerging and candidate FAD genes, as well as inherited risk factors will be also discussed, suggesting that enlarged genetic testing may be useful in FAD families in order to improve the identification and management of the at-risk subjects.

## 2. Methods

**Figure 2:**
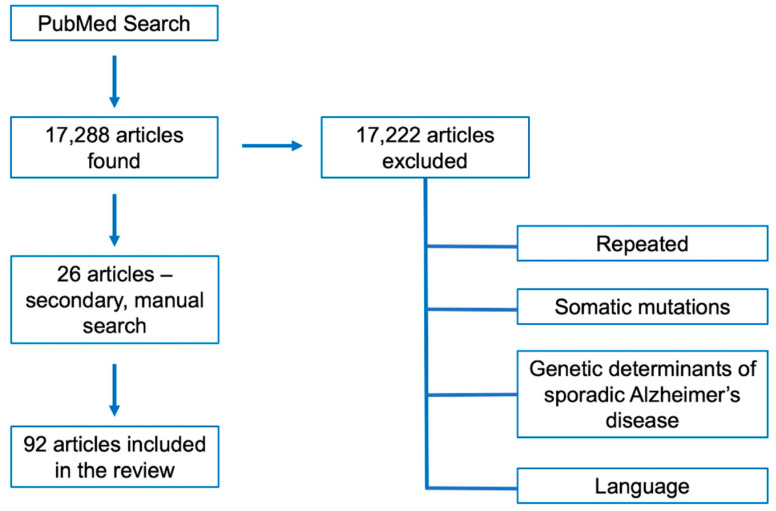
Flow-diagram summarizing the steps for the selection of the articles reviewed herein.

Indexed articles in English were searched in PubMed using the following keywords: “Alzheimer’s disease molecular bases”, “Alzheimer’s disease mutations”, “Alzheimer’s disease germline mutations”, “Alzheimer’s disease genes”, “inherited Alzheimer’s disease”, “familial Alzheimer’s disease”, “*APP* mutations”, “*PSEN1* mutations”, “*PSEN2* mutations” and “novel Alzheimer’s disease genes”. In the attempt to focus on the most recent and updated papers on these topics, we fixed the time within 2010 and 2020 as temporal window; however, a manual search for oldest references mentioned in the found articles was also carried out. Papers in the search results reporting somatic mutations or describing genetic risk factors related to sporadic, late onset AD were not included since are out of the topic of the present review (Figure 2).

## 3. Highly Penetrant Familial Alzheimer Disease-Causing Genes

**Figure 3:**
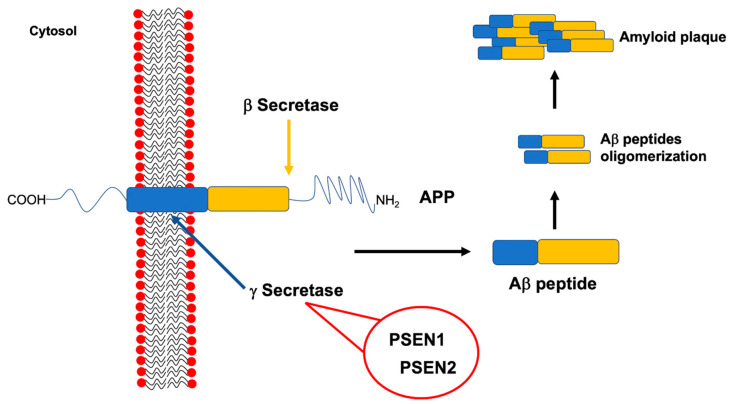
Amyloid precursor protein (APP) structure and amyloid beta (Aβ) peptide production. The cleavage of APP by specific secretases is required to produce the Aβ peptide. One of these secretases, namely the γ secretase, is a multimeric complex involving also presenilin 1 (PSEN1) and presenilin 2 (PSEN2). Mutations affecting *APP*, as well as *PSEN1* or *PSEN2* genes, have been associated to familial Alzheimer’s disease (FAD) because of their ability to increase Aβ peptides production and, consequently, their aggregation up to amyloid plaques formation.

Genetic analysis of large FAD families allowed the discovery of the three well established and high-penetrant genes related to this disease, namely, *APP*, *PSEN1* and *PSEN2* (Figure 3).

The amyloid precursor protein gene (*APP,* OMIM #104760, chromosome 21q21.3) encodes for an integral type 1 membrane glycoprotein that is almost ubiquitously expressed. APP is sequentially processed for proteolytic cleavage to produce amyloid β (Aβ), by β- and γ-secretases (the latter composed by PSEN1, PSEN2, Nicastrin and Aph-1). Because APP processing by γ-secretase is not restricted to a single site, it gives rise to different Aβ species, Aβ_42_ being more prone to aggregate [15].

Recently it has become evident that the AD-typical Aβ assemblies are able to adopt alternative conformations and become self-propagating, like prions [16,17,18,19].

To date, 32 pathogenic mutations in *APP* were reported within or flanking the Aβ sequence (https://www.alzforum.org/mutations/app), being located mostly near the β- and γ-secretase sites [20]. As a consequence, *APP* mutations seem to be able to increase Aβ aggregation rate, thus causing FAD (Figure 3) [21].

The p.K670N and p.M671L Swedish AD-related pathogenic mutations are localized near to the γ-secretase site and lead to increased absolute levels of Aβ_42_ (without changing Aβ_42_ to Aβ_40_ ratio) [22]. Interestingly, Del Prete et al., found that in a Swedish cell culture model of AD, APP and its catabolites are present in mitochondrial-ER associated membranes (MAMs) and β- and γ-secretases harbor APP processing activities in MAMs [23]. This finding is extremely interesting, considering the fact that the localization of APP and its enzymes plays a critical role in the Aβ generation and its signaling inside the cell [24]. The p.T714I, p.V715M, p.V715A, p.I716V, p.V717I and p.V717L *APP* mutations are all located in proximity of the γ-secretase site, affect the cleavage and, contrary to the double Swedish mutations, cause an increase of the Aβ_42_ to Aβ_40_ ratio and are able to influence the stability of APP C-terminal fragments [20,25]. Very recently, the p.I716T *APP* mutation [25], as well as the p.L723P featured by the local unfolding of the C-terminal turn [26], was described to affect the efficiency of the γ-secretase ε-cleavage and to induce a major Aβ_42_ to Aβ_40_ ratio. This effect is due to the additional H-bond between the T716 side chain and the transmembrane backbone, which can affect the cleavage domain dynamic [25].

Mutations within the Aβ sequence have been predicted not only to affect APP processing, thus regulating the amount of total Aβ production, but were also thought to affect the aggregation properties of the resulting Aβ peptide [25]. Indeed, the *APP* p.A692G, p.E693Q and p.D694N mutations, all located inside the Aβ sequence, have been described to have an increased aggregative ability and neurotoxicity respect to the wild type Aβ [21].

Although a common effect of these mutations is the dysregulation of the production of different Aβ forms of APP, a recent paper form Lumsden et al. proposed also a dysregulation of iron homeostasis as a common effect of mutations related to early onset AD [27].

Table 1 summarizes *APP* mutations, affecting protein stability, folding, processing, from the N- to the C-terminal.

Despite the above, it has been reported that *APP* dominant mutations account for about 16% of all ADAD [3]. Some years ago, 2 *APP* mutations, namely p.A673V and p.E693del, have been found to be able to cause FAD only in the homozygous status supporting the existence also of a recessive pattern of inheritance [28,29]. More recently, Conidi et al., described an Italian family carrying the *APP* p.A713T in homozygous status; surprisingly the clinical phenotype was not more severe respect to the heterozygous carriers [30]. This finding not only highlighted that the homozygosity for *APP* dominant mutations is not lethal, but also suggested that other, independently inherited genetic factors, may exert a protective effect and modify the clinical presentation of the disease.

Finally, in addition to single nucleotide variants and small insertion/deletions, dominantly inherited duplications of the *APP* locus have been also described and associated to AD [31,32,33,34,35]. In particular, small chromosomal duplications with different genomic coordinates, but all including the *APP* locus, have been described in some French FAD families [31,32]. Next, *APP* duplications were reported also in Finnish and Dutch FAD cases [33,34,35]. The clinical consequences of these duplications are not yet clearly defined and also their frequency as FAD cause is variable in the different studies [31,32,33,34,35]. Totally, 25 duplications have been identified so far and, respect to missense mutations, these duplications seem to have a reduced penetrance and a variable age of onset [36].

Interestingly, Jonsson et al., identified a rare *APP* variant in the Icelandic population showing a protective effect [37]. Finally, a number of variants of unknown clinical significance have been also detected and further functional tests are required to establish their pathogenicity.

The presenilin 1 (*PSEN1*, OMIM #104311, chromosome 14q24.3) encodes for a protein that is a subunit of γ-secretase, i.e., one of the 2 enzymes responsible for APP proteolytic cleavage. As a consequence, mutations in *PSEN1*, impairing the activity of γ-secretase complex, may lead to the production of more aggregation-prone forms of the Aβ peptide, that is a typical hallmark of AD, thus inducing the disease development (Figure 3) [3,4].

*PSEN1* is the most common gene related to FAD. To date, 221 *PSEN1* pathogenic mutations have been described, accounting for up to 70% of ADAD cases (http://www.alzforum.org/mutations). These mutations can be both single nucleotide variants and small insertions/deletions; in addition, a deletion able to cause *PSEN1* exon 9 skipping, has been also described [38]. Typically, FAD onset in *PSEN1* mutations carriers ranges from 30 to 50 years, the mutations showing an autosomal dominant inheritance and almost always a complete penetrance [39]. Interestingly, *de novo* mutations, featured by a very early age of onset (before 30 years) have been also described [40,41,42].

As in the case of *APP*, Kosik et al., described a Colombian family carrying the *PSEN1* mutation p.E280A in homozygous status; also, in this case, the severity of the disease was not influenced by the homozygosity of the mutation [43].

The presenilin 2 (*PSEN2*, OMIM #600759, chromosome 1q31-q42) gene has a genetic structure very similar to *PSEN1*, sharing a sequence homology of 67% [3]. It encodes for another component of the γ-secretase complex; thus, its impairment, due to pathogenic mutations, is able to increase the Aβ_42_/Aβ_40_ ratio too (Figure 3).

To date, 19 different *PSEN2* pathogenic mutations have been reported (http://www.alzforum.org/mutations). As for *PSEN1*, these pathogenic mutations are scattered along the entire gene sequence with a higher frequency in the transmembrane domains [44,45]. The ADAD age of onset ranges from 40 to 70 years in *PSEN2* mutations carriers, the penetrance of these mutations being still controversial to assess, due to the few numbers of families reported to date and showing also a so wide age-range of onset [39].

It has been estimated that totally, *APP*, *PSEN1* and *PSEN2* mutations account only for about 5–10% of all FAD (Figure 1) [46,47]. Within the FAD, considering only the ADAD forms the contribution of these genes is highly heterogeneous based on the population studied, 23% up to 88% of patients remaining without a genetic diagnosis [47,48]. Since the clinical features of FAD can be variable, the diagnosis is often difficult and delayed, underlying the importance of identifying other molecular alterations responsible for the currently unexplained FAD cases.

## 4. Apolipoprotein E ε4 Risk Allele and Familial Alzheimer Disease

The apolipoprotein E gene (*APOE*, OMIM #107741, chromosome 19q13.2) encodes a glycoprotein involved in the mobilization of peripheral cholesterol, also during neuronal growth and regeneration [49,50]. Three APOE isoforms are known, namely ApoE2, ApoE3 and ApoE4, differing at level of 2 aminoacidic residues (the 112 and 158) and coded by 3 alleles, i.e., ε2, ε3 and ε4, whose frequency varies among different populations [3].

**Figure 4:**
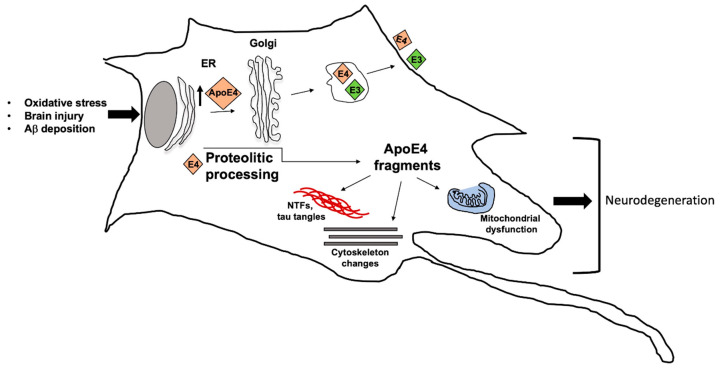
Working model for Apolipoprotein E4 (ApoE4) contribution to Alzheimer’s disease (AD) development. Different stimuli can induce ApoE4 and ApoE3 overexpression, with E4 contribution to neurodegeneration in AD. ApoE4, undergoing to proteolytic cleavage, can generate different fragments which can contribute to mitochondrial dysfunction, cytoskeletal disorganization, neurofibrillary tangles and, consequently to neurodegeneration.

To date, an association between the ε4 allele and the sporadic, late-onset AD has been reported [3,4]. In particular, it has been assessed an increased risk up to 3-fold in the heterozygous carriers and up to 15-fold in the homozygous [51]. Interestingly, the ε2 allele has been reported as a protective factor, reducing the AD risk and also positively impacting longevity [52]. These different features have been related to ta different binding affinity of the encoded proteins for the Aβ peptide; in particular, ApoE4 shows the highest affinity leading to the creation of monofibrils that are able to produce dense precipitates [3]. However, it is important to underline that the ε4 allele is not a cause but an AD risk factor; thus, other genetic or environmental factors are required for disease development. A working model, attempting to explain the relationship between ApoE and Alzheimer’s disease, has been proposed (Figure 4) [53].

In particular, ApoE4, unlike ApoE3, contributes to AD by interacting with different factors through various pathways. In response to oxidative stress, aging, brain damage or Aβ deposition, neurons synthesize increasing amount of ApoE, that in turn undergoes proteolytic processing generating fragments which cause mitochondrial dysfunction, cytoskeletal changes, NFT (neurofibrillary tangles) formation, leading to neurodegeneration.

Interestingly, it has been shown that the *APOE* ε4 allele is able to increase also the risk for early onset AD in presence of familiarity for the disease [54]. In particular, in the ε4 homozygous carriers the risk was independent from other genetic factors, while in the heterozygous no, suggesting that it may act as disease phenotype-modifier in presence of other genetic mutations [4]. However, Genin et al., reported for *APOE* ε4 allele an AD risk comparable to that of other genetic factors [55]. Some studies, evaluating the effects of the *APOE* genotype on AD clinical features in families carrying pathogenic mutations in *APP*, *PSEN1* or *PSEN2* genes, showed that the ε4 allele is associated to an earlier age of onset in the mutations’ carriers, while the carriers of the ε2 allele had a later onset [56,57,58]. Nevertheless, it is important to underline that the significance of *APOE* testing in clinical practice is still under debate and it has been recently reviewed to not significantly impact diagnostic and prognostic evaluations [59]. Indeed, being a risk factor, the *APOE* ε4 allele is common in the general population, i.e., also in healthy individual without a positive family history of AD. Further large longitudinal studies are required to assess the contribution of *APOE* to AD risk and its possible use in clinical routine settings.

## 5. Novel, Emerging and Candidate Genes Associated to Familial Alzheimer Disease

Mutations in *APP*, *PSEN1* and *PSEN2* genes, as well as the *APOE* ε4 risk allele, explain only a small percentage of all FAD cases, suggesting that other genes may play a role. In the last years, NGS based studies, through the analysis of large pedigrees, are allowing the detection of novel genes potentially related to FAD.

Guerreiro et al., analyzing a Turkish FAD family, identified a pathogenic mutation in the *NOTCH3* gene [60]. Interestingly, the same mutation was previously associated with a dementia disorder similar to AD and the proband belongs to a consanguineous family with a complex history of neurological disorders [60]. *NOTCH3* (OMIM# 600276, chromosome 19p13.12) encodes a transmembrane receptor involved in cellular signaling and embryonic development. More than 130 mutations have been reported in this gene and related to the rare syndrome cerebral arteriopathy autosomal dominant with subcortical infarcts and leukoencephalopathy; a role also in FAD has been recently proposed [61].

The finding of a shared gene between degenerative and vascular dementias suggests the presence of a similar neurovascular unit dysfunction. Accordingly, a consensus paper by Bordet et al., based on the observation that most of patients currently seem to be affected by mixed forms, proposed that also therapeutic strategies should be common [62]. Indeed, therapeutic approaches should be oriented towards an integrated strategy, including antioxidants, anti-inflammatory, modulation of proteins aggregation and neuronal plasticity. Since in older patients, vascular cognitive impairment (VCI) leading to vascular dementia is often mixed to AD and VCI is rarely “pure”, a disease modifying strategy seems to be justified [62]. It is noteworthy that a mitochondrial dysfunction may play a role in this context as an additional cause of cognitive impairment, either of vascular, degenerative or both natures [63]. In particular, a reduction of respiratory chain complex I activity, related to mitochondrial dysfunction, has been reported in a group of patients with vascular dementia and several mitochondrial mechanisms have been invoked in Aβ-related cerebrovascular degeneration [63].

Pottier et al., carried out the WES of 29 probands from FAD families resulted negative for mutations in the 3 main FAD genes and found 7 mutations in the *SORL1* gene [64]. In particular, one of these mutations, i.e., the p.G511R, has been shown to be able to reduce the ability of the protein to bind the Aβ peptide, thus inducing its accumulation [65]. The sortilin-related receptor (*SORL1*, OMIM# 602005, chromosome 11q24.1) encodes for a mosaic protein that is the receptor of neuronal ApoE. Accordingly, *SORL-1* mutations have been described in 2 families with early onset AD. In particular, the *SORL-1* variants where shown to be able to weaken the interaction with APP, interfering with APP trafficking and altering the Aβ levels [66]. Other studies have confirmed the role of *SORL1* mutations in FAD and also in late onset AD [67,68]. Li et al., have recently reported the case of a patient with early onset AD and cognitive impairment, carrying a heterozygous mutation in the *SORL1* gene [69]. Taken together, these items of evidence suggest that *SORL1* mutations may be involved in FAD, its contribution being probably underestimated, and that this gene should be tested in the affected families in addition to *APP*, *PSEN1* and *PSEN2* genes. Indeed, the analysis of *SORL1* in larger cohorts of patients may allow to better clarify its contribution to FAD.

Interestingly, genome-wide association studies identified about 30 additional risk factors/alleles for late onset AD [70,71]. Among these, variants affecting *CLU* (*APOJ*) or *CR1* (complement component 3b/4b receptor 1), being involved in the clearance of Aβ have been associated to AD [72] and heterozygous missense mutations in *TREM2* (triggering receptor myeloid 2 cells) have been described to increase by 3-fold the risk of AD [73]. It has been proposed that these genes may be responsible also for FAD cases.

In particular, an increased frequency of *CLU* gene rare coding mutations has been highlighted in AD patients, predominantly affecting the β chain of the protein [74]. Clusterin (*CLU*, OMIM# 185430, chromosome 8p21.1) encodes a protein involved in synapsis turnover. Most of the CLU variants described so far are able to promote CLU degradation, thus reducing its activity [75].

Two NGS-based studies identified a rare variant (p.Arg47His) in *TREM2* gene [73,76] *TREM2* (OMIM# 605086, chromosome 6p21.1) encodes a type I transmembrane protein belonging to the immunoglobulin receptor superfamily and involved in immune responses activation. Interestingly, TREM2 has been found to be able to bind ApoE, thus increasing the phagocytosis of ApoE-bound apoptotic neurons [77]. Some *TREM2* variants may increase AD risk by reducing the affinity for ApoE, and thus decreasing Aβ peptide clearance. Additionally, it has been showed that *TREM2* mutations in its extracellular domain impair protein maturation and its phagocytic activity [78,79]. The *TREM2* p.Arg47His variant has been reported in different population as associated to AD, including some cases of FAD [73,76,80,81,82]. The role of other *TREM2* variants is still poorly understood. 

Three independent studies identified loss-of-function mutations in the *ABCA7* gene in AD patients [83,84,85]. The ATP-binding cassette, subfamily A, member 7 (*ABCA7*, OMIM# 605414, chromosome 19p13.3) encodes a transporter protein able to move lipids across the membranes. It has been reported that the inhibition of ABCA7 expression is able to increase β secretase cleavage of APP, thus increasing the production of Aβ peptide [86]. In particular, Cuyvers et al., identified an *ABCA7* frameshift mutation as a founder mutation in a Belgian population, since it was detected in several FAD families showing a dominant pattern of inheritance [83].

Vardarajan et al., by sequencing 76 AD-related loci, identified a rare missense mutation in the *EPHA1* gene (p.P460L) segregating within a large Caribbean FAD family [85]. The Ephrin receptor (*EPHA1,* OMIM # 179610, chromosome 7q34-q35) encodes a tyrosine kinase receptor implicated in neuronal development.

The main features of novel FAD candidate genes are summarized in Table 2.

It is noticeable that the same genes identified as risk factors for sporadic and late onset AD, harbor also rare variants segregating with FAD. Even if data in this field are still inconclusive since they are often based on isolated findings, however they suggest that some familial cases may be due the combination of rare variants and other risk factors. Recent NGS-based screening including FAD cases, identified established risk alleles with moderate penetrance and one or more variants of uncertain significance, thus suggesting the hypothesis, in presence of no mutations in the 3 main FAD genes, of a polygenic inheritance [87,88]. The use of NGS-based methods for the analysis of large genomic regions in several patients simultaneously may provide further insights to improve the diagnosis of disorders featured by high genetic and phenotypic variability, such as AD. However, the interpretation of NGS data, due to the large number of variants of unknown significance, is often challenging and inconclusive in clinical settings [14,89].

## 6. Conclusions

AD incidence is showing an increasing trend worldwide, so its early and accurate diagnosis has become mandatory. Indeed, the earlier the diagnosis is made, the sooner the treatments may begin, the latter being an important prognostic factor to ameliorate patients’ clinical outcome. It is becoming evident that within the “AD” definition are included different entities, whose correct identification may be important to drive treatments choices. While most of AD cases are sporadic, featured by late onset and by the presence of polygenic risk factors, a small percentage of AD is familial, often featured by early age of onset and related to the presence of rare, pathogenic mutations segregating within the affected families. To date, 3 main genes (*APP*, *PSEN1* and *PSEN2*) have been related to autosomal dominant FAD, accounting just for a small percentage of cases. Novel candidate genes are being identified. Larger studies on large group of patients are required to better address their contribution to the disease and discover other potential candidates. This will allow a better prognostic classification of the patients and a better management of the probands and of their families, by the identification of the -at risk individuals. These genetic data, together with novel clues coming from other “omics” [90,91,92], will allow also the development of novel and even more personalized therapies for AD and FAD treatment.

## Figures and Tables

**Table 1:** Amyloid precursor protein (*APP*) pathogenic mutations and their effects at protein level.

Mutation and Protein Region	Protein Stability/Folding/Processing	Reference
*N-terminal*		
p.K670N/p.M671L	Leads to increased absolute levels of Aβ_42_, doesn’t alter Aβ_42_/Aβ_40_ ratio Present in MAMs	Kumar-Singh_2009 [22] Del Prete_2017 [23]
*Amyloid-beta domain*		
p.A692G		Murakami_2002 [21]
p.E693Q p.E693K p.E693G	Potent aggregative	
p.D694N		
p.A713T p.T714I	Increase Aβ_42_/Aβ_40_ ratio, affect stability of *APP* CTFs	Kumar-Singh_2009 [22]
*Transmembrane/C-terminal*		
p.V715M p.I716V p.V717L	Increase Aβ_42_/Aβ_40_ ratio, affect stability of *APP* CTFs	De Jonghe_2001 [20]
Pp.I716T	Increases Aβ_42_/Aβ_40_ ratio reducing the efficiency of the γ-secretase ε-cleavage	Götz_2019 [25]
p.L723P	Causes unfolding of C-terminal turn of *APP* TM domain helix	Bocharov_2019 [26]

**Table 2:** Novel candidate genes and inherited risk factors associated to familial Alzheimer’s disease (FAD).

Gene Name (Acronym)	Proposed Function in FAD	References
Apolipoprotein E (*APOE*)	Contribution of ApoE4 to mitochondrial dysfunction, cytoskeletal disorganization and neurofibrillary tangles	Huang_2006 [53]
Neurogenic Locus Notch Homolog Protein 3 (*NOTCH3*)	Cellular signaling impairment	Patel_2019 [61]
Sortilin-related receptor (*SORL1)*	Interference with APP trafficking and alteration of the Aβ levels	Cuccaro_2016 [66]
Complement component 3b/4b receptor 1 (*CR1*)	Aβ peptide clearance reduction	Shen 2016 [72]
Clusterin (*CLU*)	Synapsis turnover reduction	Bettens_2015 [75]
Triggering Receptor Expressed on Myeloid Cells 2 (*TREM2*)	Aβ peptide clearance reduction	Bailey_2015 [77] Lue_2015 [78] Kleinberger_2014 [79]
The ATP-binding cassette, subfamily a, member 7 (*ABCA7*)	Aβ peptide production increase	Satoh_2015 [86]
Ephrin receptor (*EPHA1)*	Alteration of neuronal development	Vardarajan_2016 [85]
